# Meta-analysis of *Arabidopsis thaliana* microarray data in relation to heat stress response

**DOI:** 10.3389/fpls.2023.1250728

**Published:** 2023-12-19

**Authors:** Zohra Chaddad, Kaoutar Kaddouri, Abdelaziz Smouni, Mustapha Missbah El Idrissi, Kaoutar Taha, Ichrak Hayah, Bouabid Badaoui

**Affiliations:** ^1^ Centre de Biotechnologies Végétales et Microbiennes, Biodiversité et Environnement, Faculty of Sciences, Mohammed V University in Rabat, Rabat, Morocco; ^2^ African Sustainable Agriculture Research Institute (ASARI), Mohammed VI Polytechnic University (UM6P), Laâyoune, Morocco

**Keywords:** *Arabidopsis thaliana*, heat stress, meta-analysis, microarray, transcription factor, biological process, differentially expressed genes (DEGs), gene ontology (GO)

## Abstract

**Introduction:**

Increasing global warming has made heat stress a serious threat to crop productivity and global food security in recent years. One of the most promising solutions to address this issue is developing heat-stress-tolerant plants. Hence, a thorough understanding of heat stress response mechanisms, particularly molecular ones, is crucial.

**Methods:**

Although numerous studies have used microarray expression profiling technology to explore this area, these experiments often face limitations, leading to inconsistent results. To overcome these limitations, a random effects meta-analysis was employed using advanced statistical methods. A meta-analysis of 16 microarray datasets related to heat stress response in *Arabidopsis thaliana* was conducted.

**Results:**

The analysis revealed 1,972 significant differentially expressed genes between control and heat-stressed plants (826 over-expressed and 1,146 down-expressed), including 128 differentially expressed transcription factors from different families. The most significantly enriched biological processes, molecular functions, and KEGG pathways for over-expressed genes included heat response, mRNA splicing via spliceosome pathways, unfolded protein binding, and heat shock protein binding. Conversely, for down-expressed genes, the most significantly enriched categories included cell wall organization or biogenesis, protein phosphorylation, transmembrane transporter activity, ion transmembrane transporter, biosynthesis of secondary metabolites, and metabolic pathways.

**Discussion:**

Through our comprehensive meta-analysis of heat stress transcriptomics, we have identified pivotal genes integral to the heat stress response, offering profound insights into the molecular mechanisms by which plants counteract such stressors. Our findings elucidate that heat stress influences gene expression both at the transcriptional phase and post-transcriptionally, thereby substantially augmenting our comprehension of plant adaptive strategies to heat stress.

## 1 Introduction

In recent years, global warming has emerged as a critical consequence of climate change, and it poses a significant threat to crop productivity worldwide (Abbas et al., 2018). The rising temperatures, which often surpass the optimal tolerance range for plants, result in what’s known as heat stress -a major abiotic stressor. This condition significantly affects plant growth and overall agricultural output (Li et al., 2011). Heat stress induces oxidative stress and ultrastructural alterations in various plant parts, causing membrane fluidization, lipid bilayer disintegration (Los et al., 2013), unsaturated fatty acid peroxidation, and the promotion of reactive oxygen species (Boca et al., 2014). These affect photosynthesis and nutrient uptake and reduce plant growth and yield (Ali et al., 2020).

To mitigate future risks to global food security, the development of heat-tolerant crops with enhanced productivity holds great promise. Understanding the physiological, molecular, and genetic mechanisms that govern the response to heat stress in model plants is of great value. It can offer insights into improving heat stress tolerance in other plant species, including important agricultural crops (Singh et al., 2019). Consequently, it is crucial to investigate potential mechanisms enabling plants to respond to heat stress and identify genes involved in this response. Despite extensive use of transcriptional profiling assays to identify heat stress-related genes and potential tolerance-inducing mechanisms, there remains a substantial unexplored territory regarding signaling pathways, plant hormones, and transcription factors (TFs) associated with heat stress response (Zhao et al., 2020). Furthermore, the outcomes of these studies often exhibit inconsistencies and fail to fully capture the real-world heterogeneity due to variations in transcript levels resulting from environmental conditions and plant development. Additionally, the high cost of analysis often limits the number of repetitions considered in studies, typically allowing only two (Haynes et al., 2017; Zhang et al., 2017).

Meta-analysis represents a potent approach that effectively mitigates the limitations often encountered in individual expression profiling studies. It plays a pivotal role in enhancing the reproducibility and reliability of results by enhancing statistical power for detecting expression changes, thus providing a more robust and precise identification of differentially expressed genes (DEGs) (Haynes et al., 2017). Microarray technology has been extensively employed to investigate the heat stress response in *Arabidopsis thaliana*, producing vast datasets amenable to meta-analyses. These meta-analyses are important in the quest to pinpoint key genes and elucidate the mechanisms vital to the plant’s response to heat stress. In this context, this study is dedicated to the identification of genes exhibiting both upregulation and downregulation in response to heat stress. Furthermore, we explored how biological processes, molecular functions, and pathways are affected by the identified DEGs in both upregulated and downregulated directions.

## 2 Materials and methods

### 2.1 Dataset collection and processing

In this study, the Gene Expression Omnibus (GEO) (Edgar et al., 2002) and ArrayExpress (AE) (Brazma et al., 2003) databases were utilized to select *A. thaliana* expression profiling datasets related to heat stress conditions. Searches were conducted using the keywords “heat stress“, “heat shock“, and “abiotic stress“ and filtering results by “*Arabidopsis thaliana*“ and “Expression Profiling by Array“. Abstracts and keywords of the datasets were carefully examined, and only datasets meeting all the following criteria were used for meta-analyses:

- Dataset derived from mRNA expression profiling using single-channel microarray technology: Single-channel microarrays are widely used for gene expression profiling, making it easier to combine and compare data from different sources.
- Probe-gene mapping annotation from the Affymetrix platform [http://www.affymetrix.com/technology/index.affx]: It is a well-established and reputable microarray platform. Its use allows for consistent annotation and interpretation of gene expression data and reduces platform-specific complications.
- At least two controls and two case samples: The presence of multiple replicates in each dataset allows for assessing the heterogeneity of effects across datasets.
- Control samples originated from plants not exposed to heat stress or any other stress, while case samples originated from plants exposed solely to heat stress.
- Processed gene expression data: To reduce the data complexity and ensures data consistency across the selected datasets.

Each dataset was manually curated to exclude samples exposed to other treatments than heat stress, even in combination. The random-effects meta-analysis was used to account for the presence of heterogeneity, including factors such as light intensity, humidity, recovery time, and plant age , allowing the combination of different studies (Borenstein et al., 2009). To find out how mutant samples affected the different results, two meta-analyses were done: one with all the chosen samples, and the other with only wild-type A. thaliana samples, leaving out the mutant samples.

We downloaded the expression data and all available annotations for the selected datasets from AE database. The GEO datasets were automatically obtained using the MetaIntegrator package (Haynes et al., 2017). Classes (1 for heat stressed samples and 0 for control samples) were manually assigned for each dataset. For all selected datasets, the normalization was unnecessary, as the median values of the samples were similar within each dataset, and the data was already in log scale due to expression values ranging from 0 to 15.

### 2.2 Meta-analysis and differentially expressed genes identification

**Figure 1:**
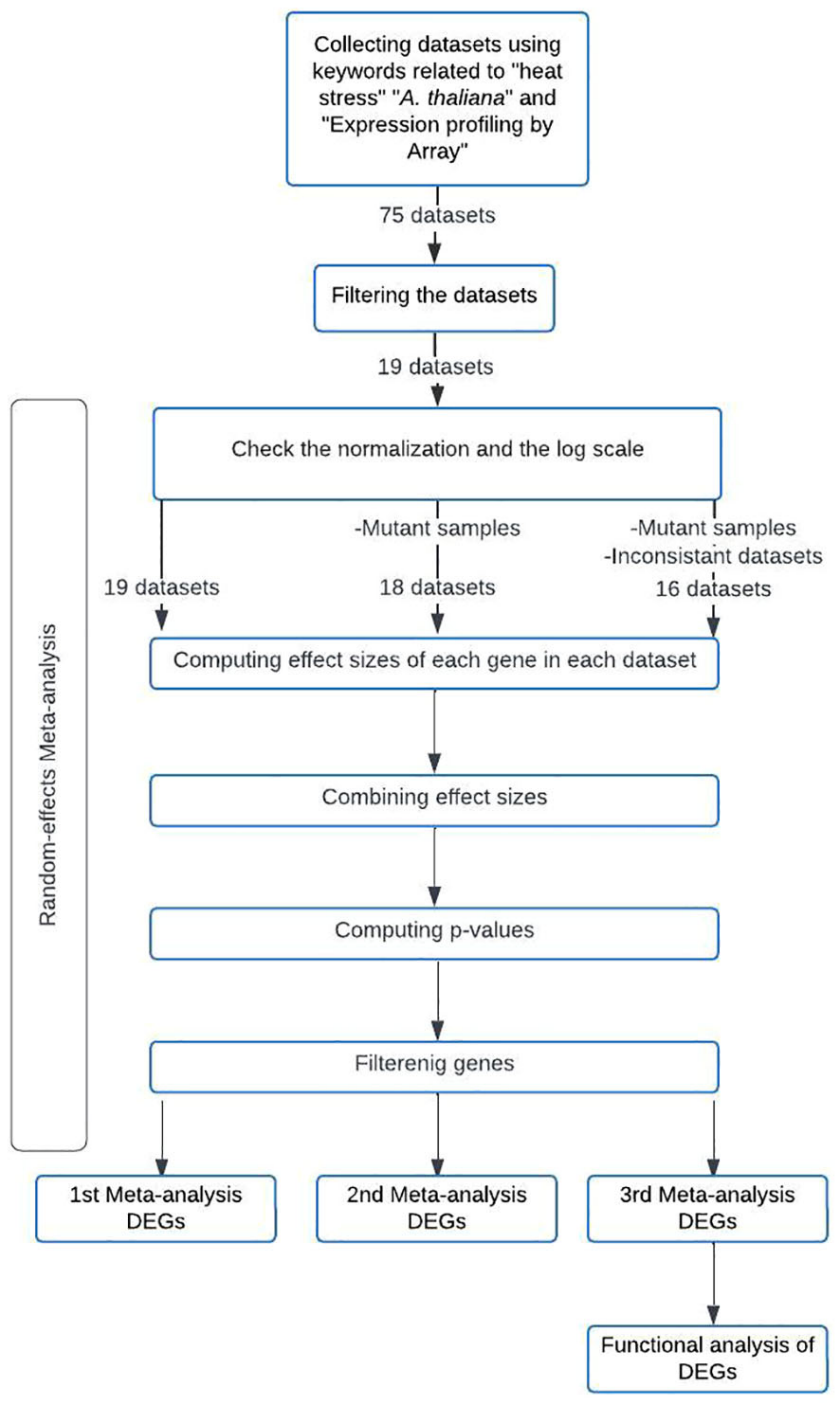
Methodology Flowchart for the Conducted Meta-Analyses.

A flowchart was created to summarize the meta-analysis methodology employed in this study (**Figure 1**). The meta-analysis of the selected microarray datasets was conducted using the MetaIntegrator R package. Hedges’ g effect size (Borenstein et al., 2009) was calculated for each gene in each dataset to determine the effect size (ES). The computed ESs were combined using a random-effects model with the inverse-variance method to obtain the summarized effect size (SES). The p-value for each gene was calculated using z-statistics based on a standard normal distribution, using the SES and its corresponding standard error (Khatri et al., 2013). To minimize false-positive results, p-values were adjusted for multiple hypothesis testing using the Benjamini-Hochberg False Discovery Rate (FDR) correction (Benjamini and Hochberg, 1995). Cochran’s Q value was also calculated to assess the heterogeneity of the ES estimates between datasets. Cochran’s Q p-value was computed and adjusted with the Benjamini-Hochberg FDR correction (Haynes et al., 2017).

DEGs involved in the heat stress response were identified using the FilterGenes function in the MetaIntegrator R package by adjusting filtering parameters. The cutoffs for the absolute value and the false discovery rate (FDR) of the SES were 1 and 0.001, respectively. The gene must show consistent over- or under-expression across all included datasets to be included in the DEGs list. To examine the heterogeneity of gene expression among different selected datasets, the heterogeneity of the ES was chosen as a cutoff of 0 to retain heterogeneously expressed genes and a cutoff of 0.05 to remove all significantly heterogeneous genes.

### 2.3 Visualizing and validating the resulting DEGs

The performance of the identified DEGs in the conducted meta-analyses was evaluated to differentiate between heat-stressed samples and control samples in each dataset. This evaluation involved validation of specificity and sensitivity using Receiver Operating Characteristic (ROC) curves and Area Under the ROC Curve (AUC) measurements, facilitated by the MetaIntegrator R package. To display the ES of the obtained DEGs across different datasets and offer an overview of the expression profiles of the selected DEGs in all datasets, a heatmap was generated using the MetaIntegrator R package.

### 2.4 Gene ontology annotation and pathway analysis

GO terms facilitate an understanding of the fundamental biological processes and molecular functions mediated by genes. The g:Profiler database (Raudvere et al., 2019) was used to perform GO enrichment analysis for both over-expressed and down-expressed genes with a significant p-value < 0.05 to uncover significantly enriched biological processes and molecular functions.

The identified DEGs were converted to TAIR-LOCUS using Gene ID conversion in the g:Profiler platform, as the Affymetrix Arabidopsis ATH1 Genome Array [ATH1-121501] uses open reading frames (ORFs) to map probe sets. Unknown ORFs in this database were manually matched using available information from the GPL198 platform in the GEO database.

The REVIGO database (Supek et al., 2011) and Treemap R package were employed to summarize extensive and complex lists of biological process GO terms by identifying a representative subset of these terms using a clustering algorithm based on semantic similarity measures. Pathway enrichment analysis for DEGs was conducted based on the Kyoto Encyclopedia of Genes and Genomes (KEGG) using the DAVID v.6.8 database (Huang et al., 2009).

### 2.5 Identifying transcription factors

The list of *A. thaliana* TFs was obtained from the Plant Transcription Factor Database PlantTFDB v5.0 (Jin et al., 2017). The identified DEGs were then matched with the TFs list using the merge function in R to pinpoint over- and down-expressed TFs.

### 2.6 Co-expression network analysis

To identify DEGs with similar expression patterns and hub genes, we used the Search Tool for the Retrieval of Interacting Genes/Proteins (STRING) v11.0 database (Szklarczyk et al., 2019). We used co-expression evidence from the String database. This database relies on extensive gene-by-gene correlation tests from a vast array of gene expression datasets. These datasets are compiled by processing and mapping numerous experiments archived in GEO database as described by Franceschini et al. (2013).

The complete list of detected DEGs was submitted, including upregulated and downregulated genes. We set the organism to *A. thaliana* and the co-expression network analysis was conducted by setting the minimum required interaction score to the highest confidence level (0.9). Additionally, we concealed disconnected nodes within the network for enhanced clarity. Nodes represent the encoded proteins, while edges indicate significant co-expression scores. Using Cytoscape software (3.10.1), hub genes were identified based on their extensive connectivity within the network (Shannon et al., 2003).

### 2.7 Computing platform

We used high-performance computing through an account with access to the HPC-MARWAN computing cluster [https://www.marwan.ma/index.php/services/hpc] to perform all analyses. The necessary statistical calculations were conducted using the R programming language (version 3.6.2), which can be downloaded from [https://cran.r-project.org/], along with associated packages.

## 3 Results

### 3.1 Dataset selection

Using the keywords mentioned in the Materials and Methods section and filtering for *A. thaliana* and Expression Profiling by Array, we found 54 and 58 related datasets in the GEO and AE databases, respectively, with 37 found in both databases. Out of the 75 datasets, 19 met all the criteria specified in the Materials and Methods section (17 from GEO database and 2 from AE database) with the following accession numbers: GSE112161, GSE103398, GSE83136, GSE63372, GSE63128, GSE58620, GSE58616, GSE44053, GSE44655, GSE26197, GSE26266, GSE19603, GSE12619, GSE16222, GSE6154, GSE4760, GSE4062, E-MEXP-1725, and E-MEXP-98. These datasets consisted of 214 samples (including mutant samples), with 92 control samples (untreated plants) and 122 case samples (exposed to heat stress).

All selected datasets were published between 2004 and 2019 and derived from the Affymetrix Arabidopsis ATH1 Genome Array [ATH1-121501] [https://www.ncbi.nlm.nih.gov/geo/info/geo_affy.html]. The datasets included samples from whole seedlings, shoots, and leaves that ranged from 4 and 58 days of age. Control plants were maintained at temperatures between 20 and 24°C, while heat-stressed plants (cases) were exposed to temperatures ranging from 30 to 44°C for 30 minutes to 1 day, with or without recovery time whether in light or dark conditions (**Table S1**).

### 3.2 Microarray meta-analyses including/excluding mutant samples

A gene is considered differentially expressed between control and heat-stressed samples when it meets specific criteria. First, the absolute SES value must be greater than or equal to 1. Second, the SES FDR should be less than or equal to 0.001. In addition, the gene is required to show significant over- or under-expressed in all used datasets.

Two meta-analyses were performed, one including samples from mutant plants and the other excluding them (**Table 1**). In the meta-analysis that included mutant samples (encompassing 19 datasets, 218 samples with 92 controls and 126 cases), a total of 2779 differentially expressed genes were identified (1038 over-expressed and 1741 under-expressed). However, when significantly heterogeneous DEGs were removed by adjusting the heterogeneity threshold to 0.05 in the filtering parameters only 473 genes (177 over-expressed and 296 under-expressed) were detected as DEGs. In this case, 82.97% of the DEGs were found to be heterogeneous across the datasets. **Tables S2** and **S3** provide lists of DEGs (with and without heterogeneous DEGs, respectively), along with their SESs and Cochrane’s Q values with their respective FDRs.

**Table 1:** DEGs obtained under different meta-analysis conditions, with keeping or removing significantly heterogeneous genes.

Meta-analyses	Conditions	No. of datasets	No. of samples (control/case)	No. of DEGs with heterogenous genes (over/down expressed)	No. of DEGs without heterogeneous genes (over/down expressed)
1^st^ Meta-analysis	Including mutant samples	19	218 (92/126)	2779 (1038/1741)	473 (177/296)
2^nd^ Meta-analysis	Removing mutant samples	18	131 (56/75)	2008 (862/1145)	1998 (853/1145)
3^rd^ Meta-analysis	Removing mutant samples and E-MEXP-1725 and E-MEXP-98 datasets	16	123 (52/71)	1986 (838/1148)	1972 (826/1146)

In the meta-analysis excluding mutant samples (including 18 datasets, 131 samples with 56 controls and 75 cases), 2008 DEGs were identified (862 over-expressed and 1145 under-expressed). After the removal of significantly heterogeneous DEGs, 1998 DEGs remained (853 over-expressed and 1145 under-expressed). In this case, only 0.49 % of the DEGs were found to be heterogeneous across the datasets. **Tables S4** and **S5** present the lists of DEGs and all associated statistics for this second meta-analysis, both with and without heterogeneity removal. Notably, the first meta-analysis exhibited a much higher rate of heterogeneous DEGs, whereas the second meta-analysis had a very lower rate (0.49%).

### 3.3 Assessment of identified DEGs

A heatmap was created for the selected DEGs from both the first and second, meta-analyses using the MetaIntegrator R package. This visualization enabled the comparison of expression patterns for selected genes across different datasets and provided an overview of the selected DEGs’ expression profiles in all datasets. The heatmap revealed that the expression patterns of datasets E-MEXP-1725 and E-MEXP-98 were inconsistent with other datasets and the combined expression pattern. These discrepancies became more apparent after removing mutant samples (**Figure S1**).

The meta-analysis was conducted without including mutant samples, as well as excluding the E-MEXP-1725 and E-MEXP-98 datasets (comprising 16 datasets, 123 samples with 52 controls, and 71 cases). This analysis resulted in 1986 DEGs, with 838 over-expressed and 1148 under-expressed (**Table 1**). After removing significant heterogeneity, 1972 DEGs were obtained: 826 over-expressed and 1146 under-expressed. **Tables S6** and **S7** list the DEGs and their associated statistics for this third meta-analysis, both with and without heterogeneity removal.

**Figure 2:**
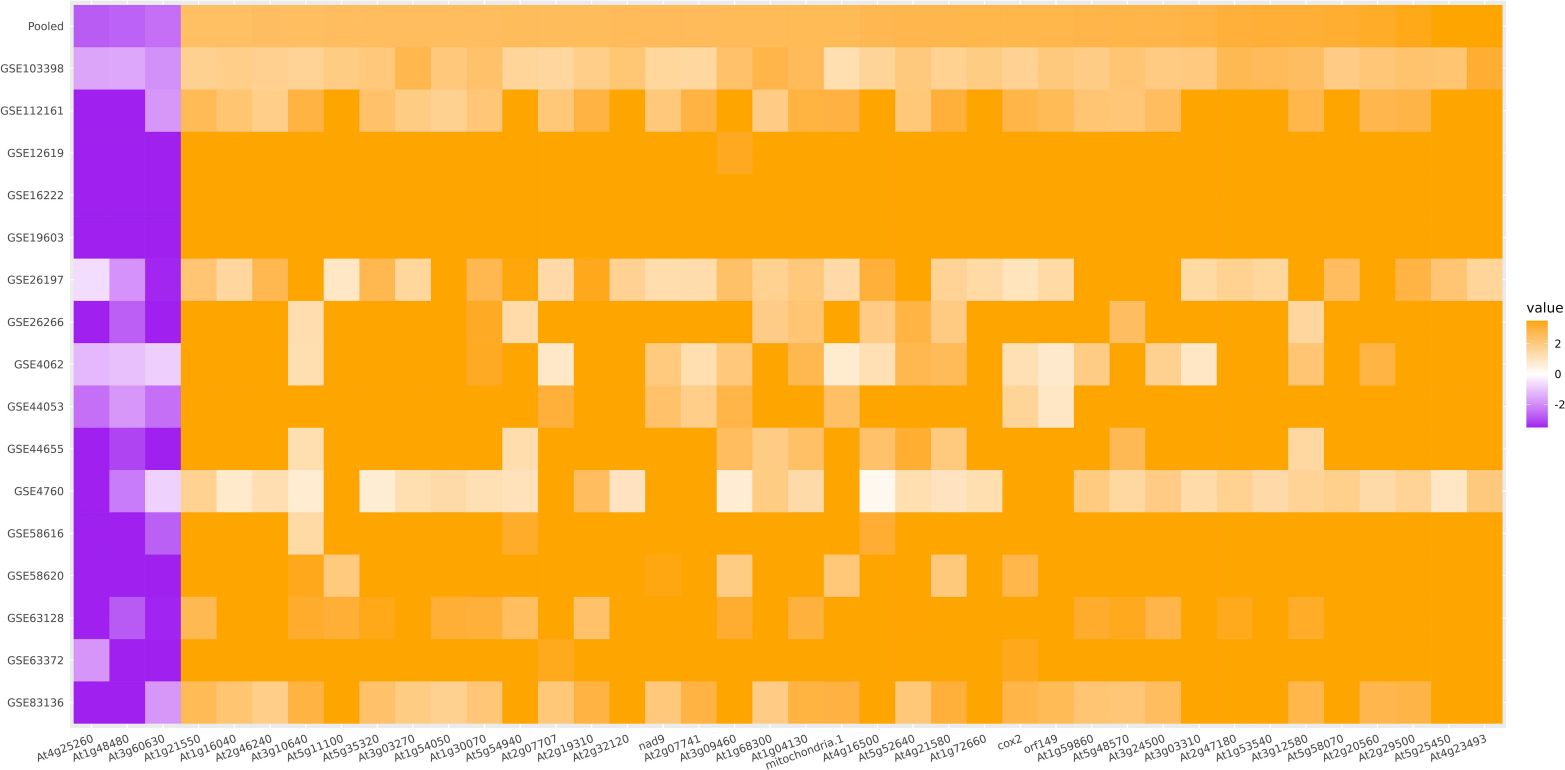
Heatmap of Prominently Upregulated and Downregulated DEGs in Meta-Analysis Across 16 Datasets (Effect Size > 2, FDR < 10^-9^, Heterogeneity Cutoff ≤ 0.05).

Given the varied nature of results stemming from the inclusion of mutant samples and the inconsistent findings in the E-MEXP-1725 and E-MEXP-98 datasets, we focused exclusively on the differentially expressed genes (DEGs) derived from the third meta-analysis. This involved excluding all mutant samples, excluding the E-MEXP-1725 and E-MEXP-98 datasets, and removing genes showing significant variability. These selected DEGs were then used for further analysis in this research. **Table S7** includes the whole list of DEGs. Additionally, **Figure S2** displays a heatmap illustrating the ES of the 1972 significant DEGs identified in the meta-analysis across the 16 selected datasets. The heatmap in the **Figure 2** illustrate the most prominently upregulated and downregulated DEGs among these 16 datasets.

**Figure 3:**
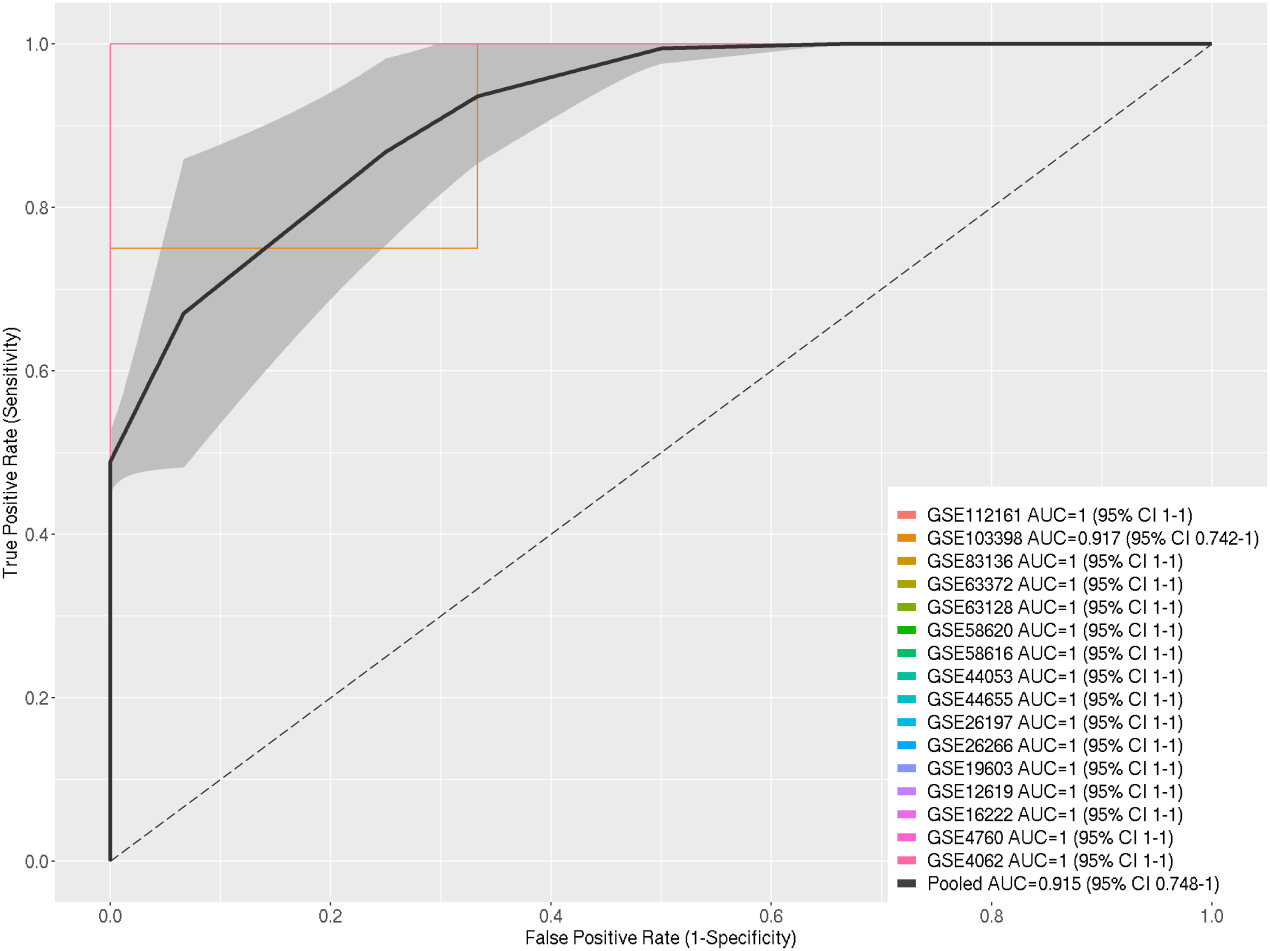
ROC Curves for Individual Datasets and Pooled ROC Curve with AUC Determination and Pooled AUC with Standard Error within 95% Confidence Interval for Selected DEGs in Response to Heat Stress in A. thaliana.

The Receiver Operating Characteristic Curve (ROC-Curve) and the pooled Area Under the Curve (AUC) were employed to evaluate the performance of the selected DEGs in discriminating between heat-stressed samples and control samples across the 16 datasets used in the meta-analysis. Out of the sixteen datasets, fifteen exhibited excellent AUC values (100%), while the remaining one demonstrated a high AUC (91.7%). The pooled AUC of the selected 16 datasets reached 91% (**Figure 3**).

## 4 Gene ontology enrichment analysis

### 4.1 Enriched biological processes

>For over-expressed genes, 109 biological processes (BPs) were significantly enriched by 826 genes. The most critical processes were: response to heat (GO:0009408), protein folding (GO:0006457), and response to temperature stimulus (GO:0009266), with p-values of 1.51×10^-34^, 1.19×10^-19^, and 3.24×10^-18^, respectively. In contrast, 69 significant BPs were enriched by 1146 under-expressed genes. The most critical among them were transmembrane transport (GO:0055085), carbohydrate metabolic process (GO:0005975), and cell wall organization or biogenesis (GO:0071554), with p-values of 2.24×10^-12^, 5×10^-10^, and 2.02×10^-8^, respectively (**Tables S2**, **3**).

**Figure 4:**
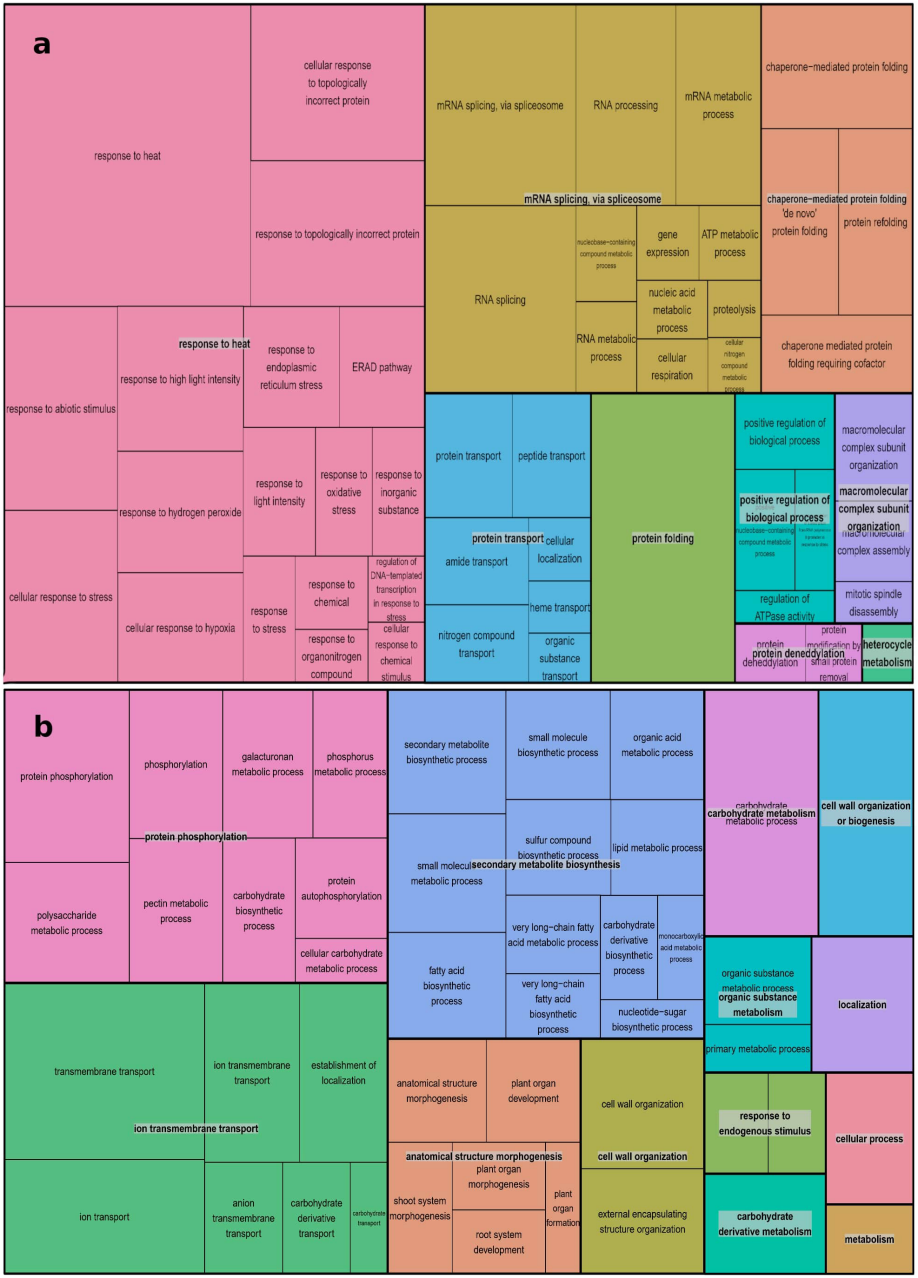
REVIGO Treemap Analysis of Biological Process GO Terms for Over-Expressed **(A)** and Down-Expressed **(B)** Genes in A. thaliana Using Treemap R Package: Clustering Algorithm Based on Semantic Similarity Measures, Superclusters Visualization, and P-Value Representation.

The REVIGO database and Treemap R package were employed to summarize the extensive lists of BP GO terms obtained. The summarized results are presented in **Figures 4A, B**, where different colors represent superclusters and rectangle sizes are adjusted to reflect the p-value. The 109 enriched BP terms for over-expressed genes were condensed into 9 superclusters, with the most important being responses to heat (18 subsets), mRNA splicing via spliceosome (12 subsets), and chaperone-mediated protein folding (4 subsets). A similar process was conducted for the 69 BP GO terms for under-expressed genes, resulting in 13 superclusters. Protein phosphorylation, ion transmembrane transport, and secondary metabolite biosynthesis were the most represented, with 9, 7, and 12 subsets, respectively.

### 4.2 Enriched molecular functions

*We also used g:Profiler to do Molecular Function (MF) gene ontology enrichment for both over- and under-expressed genes, with a 0.05 p-value cutoff.* From the eight molecular functions enriched for over-expressed genes, unfolded protein binding (GO:0051082), heat shock protein binding (GO:0031072), and misfolded protein binding (GO:0051787) were the top three enriched functions, with adjusted p-values of 1.30×10^-13^, 6.24×10^-13^, and 2.66×10^-07^, respectively. Among the 26 enriched molecular functions for under-expressed genes, transmembrane transporter activity (GO:0022857), transporter activity (GO:0005215), and ion transmembrane transporter activity (GO:0015075) were the most enriched, with adjusted p-values of 9.82×10^-13^, 2.40×10^-11^, and 7.42×10^-09^, respectively (**Table 2**).

**Table 2:** List of molecular function GO terms enriched for the over- and down-expressed genes with a p-value <0.05 in response to heat stress in *A. thaliana*.

Molecular function GO Term	Adjusted p-value	No. of DEGs
Up regulated molecular functions		
GO:0051082: unfolded protein binding	1.30E-13	30
GO:0031072: heat shock protein binding	6.24E-13	17
GO:0051787: misfolded protein binding	2.66E-07	10
GO:0003723: RNA binding	9.03E-05	82
GO:0051879: Hsp90 protein binding	0.000295	6
GO:0051087: chaperone binding	0.00157	9
GO:0060590: ATPase regulator activity	0.003461	6
GO:0017069: snRNA binding	0.007737	7
Down regulated molecular functions		
GO:0016773Phosphotransferase activity, alcohol group as acceptor	4.82E-07	108
GO:0015291Secondary active transmembrane transporter activity	2.08E-06	45
GO:0008509Anion transmembrane transporter activity	4.86E-06	38
GO:0016740Transferase activity	2.02E-05	263
GO:0016301Kinase activity	5.67E-05	113
GO:0015293symporter activity	0.001052	23
GO:0008514organic anion transmembrane transporter activity	0.00126	24
GO:0008324cation transmembrane transporter activity	0.002719	46
GO:0022890inorganic cation transmembrane transporter activity	0.002803	44
GO:0046943carboxylic acid transmembrane transporter activity	0.003981	18
GO:0005342organic acid transmembrane transporter activity	0.003981	18
GO:1901505carbohydrate derivative transmembrane transporter activity	0.006338	18
GO:0016772transferase activity, transferring phosphorus-containing groups	0.008135	120
GO:0060089molecular transducer activity	0.010387	32
GO:0005524ATP binding	0.029816	150
GO:0036094small molecule binding	0.030196	199
GO:0015077: monovalent inorganic cation transmembrane transporter activity	0.035992	30
GO:0004674: protein serine/threonine kinase activity	0.04211	68
GO:0005338: nucleotide-sugar transmembrane transporter activity	0.044498	8

### 4.3 Enriched KEGG pathways

KEGG pathways for over- and under-expressed genes were identified using the DAVID database, with an FDR < 0.05. The top three significant KEGG pathways enriched for over-expressed genes were Ath03040: Spliceosome, Ath04141: Protein processing in the endoplasmic reticulum, and Ath03050: Proteasome, with FDR values of 1.43×10^-14^, 2.96×10^-09^, and 0.046959, respectively. Six significant KEGG pathways were identified for under-expressed genes, with the most enriched pathway being ath01110: Biosynthesis of secondary metabolites, with an FDR of 1.99×10^-4^ (**Table 3**).

**Table 3:** Enriched KEGG pathways by over and down- DEGs with an FDR < 0.05 in response to heat stress in A. thaliana.

Term	No. of DEGs	FDR
Over expressed pathways		
ath03040: Spliceosome	38	1.43E-14
ath04141: Protein processing in endoplasmic reticulum	33	2.96E-09
ath03050: Proteasome	9	0.046959
Down expressed pathways		
ath01110: Biosynthesis of secondary metabolites	71	1.99E-04
ath01100: Metabolic pathways	106	5.32E-04
ath00040: Pentose and glucuronate interconversions	11	0.026269
ath00520: Amino sugar and nucleotide sugar metabolism	15	0.026269
ath00710: Carbon fixation in photosynthetic organisms	10	0.032732
ath00062: Fatty acid elongation	7	0.033125

### 4.4 Over- and under-expressed transcription factors in response to heat stress

Identifying TFs is crucial for understanding the heat stress response mechanism in *A. thaliana*. In this species, 1717 loci encode 2296 TFs, classified into 58 families according to the PlantTFDB (Jin et al., 2017). Over and under-expressed TFs in response to heat stress were identified among the DEGs. The TF encoded by each gene was determined using STRAING v.11 (**Table 4**). From 1972 DEGs obtained through meta-analysis in response to heat stress, 128 (6.49%) genes encode TFs belonging to 35 families, with 50 over-expressed and 78 under-expressed TFs. The highest number of over-expressed TFs belonged to the Ethylene Responsive TFs family (ERF) with 9 genes, followed by the bZIP family with 8 genes, and the Heat Shock Factor family (HSF) with 7 genes. The largest number of under-expressed TFs belonged to the bHLH family with 7 genes, followed by the MYB family with 6 genes, and the ARF, MYB-related, and GRAS families, each with 5 genes. The GRAS, WRKY, G2-like, GATA, NAC, C2H2, MYB-related, C3H, NF-YB, Trihelix, ERF, and bZIP families contained both over- and under-expressed genes. Notably, only under-expressed TFs were detected in the LBD, LSD, MIKC-MADS, NF-YA, HD-ZIP, Nin-like, SBP, and ZF-HD.

**Table 4:** Over- and down-expressed TFs in response to heat stress in *A. thaliana*.

TF Family	Over-expressed TFs	Down-expressed TFs
AP2	_	ADAP, RAP2.7, SMZ, AIL6
ARF	_	MP, ETT, ARF16, ARF8, ARF4
ARR-B	_	RR14, RR12, RR10
B3	ABS2	_
BBR-BPC	BPC4	_
BES1	_	BEH1
bHLH	_	SCRM2, AT1G29950, AT3G07340, AT3G61950, MYC4, BIM1, bHLH071
bZIP	GBF4, bZIP44, EEL, ABF4, BZIP25, GBF2, AHBP-1B, OBF5	bZIP2
C2H2	HD2C, ZAT6, RHL41	ZFP7, ZFP4, IDD14, IDD5, AT4G17810
C3H	AT5G40880, AT5G51980	AT5G12850
DBB	LZF1	_
Dof	_	OBP2, DOF1, AT2G28810, DOF2
ERF	CRF7, RAP2.6, RAP2.4, DREB19, AT2G40350, DREB2B, RAP2.2, CRF6, DREB2A	AT4G16750, CRF2, AT5G07580
G2-like	AT1G49560	AT5G05090
GATA	ZML1	CGA1
GRAS	SCL14	SCL27, SCR, SCL22, HAM3, AT5G66770
HD-ZIP III	_	PHB, HB-8
HSF	HSFA2, HSFA1E, HSFA7A, AT-HSFA7B, HSF4, HSFA3, HSFB2A	_
LBD	_	LBD21
LSD	_	LOL1
MIKC-MADS	_	AGL16
MYB	_	FLP, AT1G49010, AS1, MYB30, MYB16, MYB28
MYB-related	TRFL3, AT2G13960	MYBL2, ETC2, AT3G16350, AT5G47390, AT5G58900
NAC	ATAF1, NAC13, NAC069, RD26, NTL11	NAC1, NAC083
NF-X1	NFXL1	_
NF-YA	_	NF-YA6
NF-YB	NF-YB12, NF-YB13	NF-YB2
NF-YC	NF-YC3, NF-YC2	_
Nin-like	_	AT2G17150, AT4G35270
SBP	_	SPL11, SPL9
TALE	_	BLH7, ATH1, BLH6
TCP	_	TCP3, TCP10, TCP4, TCP2
Trihelix	GT-1, AT3G10030	GTL1
WRKY	WRKY32	WRKY17, WRKY47, WRKY11
ZF-HD	_	HB34, HB23

### 4.5 Co-expression network analysis

**Figure 5:**
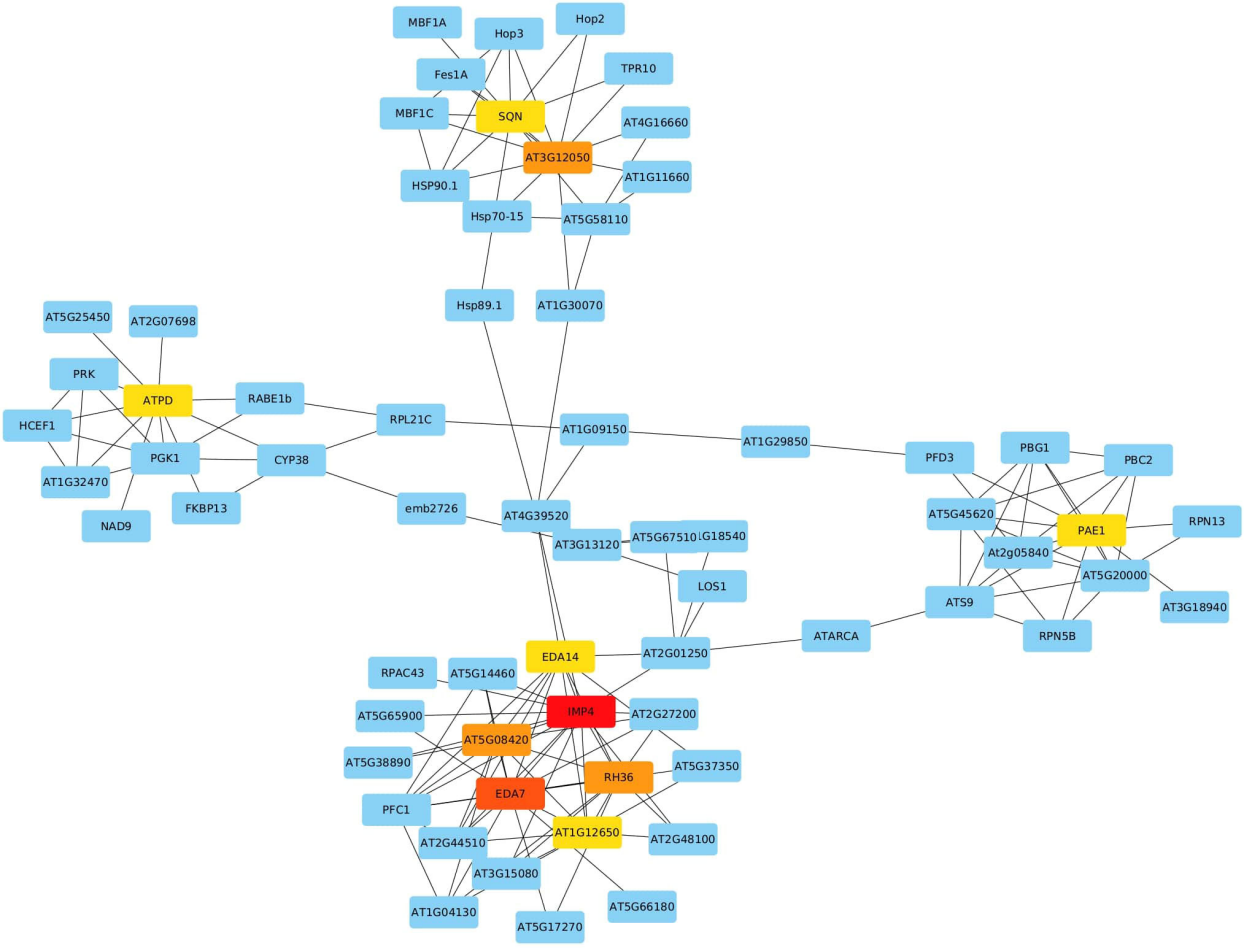
Network Analysis of Selected DEGs Identifying 10 Hub Genes (IMP4, EDA7, AT5G08420, RH36, AT3G12050, AT1G12650, ATPD, EDA14, PAE1, SQN) with High Network Connectivity.

We utilized the complete list to explore their co-expression interactions via the String v.11 platform. By setting the minimum required interaction score at the highest confidence level (0.9), we identified 266 edges connecting the 1959 submitted genes. Notably, 10 genes with a high degree of centrality emerged as hub genes using Cytoscape (v 3.10.1), indicating their extensive connectivity within the network. These hub genes include *Imp4*, *Eda7*, *At5g08420*, *Rh36*, *At3g12050*, *At1g12650*, *Atpd*, *Eda14*, *Pae1*, and *Sqn*, each scoring 16, 14, 11, 11, 11, 10, 10, 10, 10, and 10, respectively (**Figure 5**). In the network, nodes represent genes, while edges represent interactions based on co-expression evidence.

## 5 Discussion

### 5.1 Identified DEGs

There is no doubt that the analysis of genes and mechanisms involved in heat stress in plants is crucial for the development of heat-tolerant crops in the context of global warming. To this purpose, we considered that the meta-analysis using transcriptomic data is a very useful tool for several reasons. Firstly, variations in transcript levels due to environmental conditions and plant development can cause differences in gene expression across similar studies. Secondly, the high cost of analysis often limits the number of repetitions considered in studies (usually only two). Thirdly, identifying significant genes through meta-analysis of independent studies addressing the same biological question provides a statistically robust strategy (Balan et al., 2018). In this work, we aimed to highlight key biological processes, molecular functions, and pathways associated with the heat stress response in *A. thaliana* and to suggest candidate genes as heat stress biomarkers using a random effects meta-analysis.

Performing meta-analysis exclusively on wild type *A. thaliana* was the optimal approach to identify the most consistent differentially expressed genes (DEGs) since the mutant plants in this study increased result heterogeneity. This was demonstrated by comparing the meta-analysis heterogeneity of DEGs when using only wild-type samples (W) or a combination of wild-type and mutant samples (W/M). Heterogeneous DEG rates were 82.98% for W/M, while W yielded a significantly lower heterogeneity rate of 0.5% (**Table 1**). Including mutant samples introduced significant variability, which posed challenges in the identification of consistent DEGs. Excluding mutant samples allowed us to focus on the responses of wild-type plants to heat stress.

In this study, 1972 genes were identified as differentially expressed in response to heat stress treatment, with 826 (41.59%) over-expressed and 1146 (58.41%) down-expressed under the significance threshold of FDR ≤ 0.001 and an absolute value of SES ≥ 1. Furthermore, these genes exhibited significant over or down-expression without substantial heterogeneity across all 16 datasets used.

We used the Area Under the ROC Curve (AUC) to show how well the chosen DEGs could tell the difference between heat-stressed samples and control samples in the 16 datasets that made up this meta-analysis. Fifteen out of the sixteen datasets exhibited an exceptional AUC value (100%), while the remaining dataset achieved a high AUC (91.7%), leading to a combined AUC value of 91.5%. Consequently, the selected DEGs could distinguish between heat-stressed and non-heat-stressed *A. thaliana* with an extremely low probability of false positives. This indicates that the expression patterns of the DEGs can differentiate between heat-stressed and non-heat-stressed *A. thaliana* plants, validating the significant relevance of these DEGs to the heat-stress response (**Figure 3**).

Among the DEGs, 8 genes were highly over-expressed with an SES greater than 3; *At4g23493* was the most over-expressed gene (with a yet unknown function and encoded protein) and had an SES of 3.57. The other most over-expressed genes included *At5g25450*, *At2g29500*, *At2g20560*, *Til* (*AT5G58070*), *Hsp70* (*AT3G12580*), *At1g53540*, and *GolS1* (*AT2G47180*). For the down-expressed genes, *At4g25260*, *Rkl1* (*AT1G48480*), and *Scl22* (*At3g60630*) (were the top three with an SES less than -2.40. Notably, the *Til* gene exhibited strong over-expression due to its involvement in thermotolerance, potentially by inhibiting plasma membrane lipid peroxidation caused by intense heat shock. Boca et al. (2014) demonstrated that *Til* knockout *A. thaliana* is much more sensitive to heat stress than the wild type. Conversely, *At4g25260*, *At1g48480*, and *At3g60630* were the most down-expressed genes with an SES less than -2.40 (**Table S7**, **Figure 2**).

### 5.2 Enriched GO terms and TFs

In response to extreme environmental conditions, such as heat stress, plants undergo extensive transcriptomic, proteomic, and metabolic adjustments to adapt and survive. Our meta-analysis revealed the enrichment of several important pathways, molecular functions, and biological processes. As anticipated, the most significantly up-regulated processes included responses to heat, mRNA splicing via spliceosome, protein transport, chaperone-mediated protein folding, and protein folding. On the other hand, processes such as protein phosphorylation, secondary metabolite biosynthesis, anatomical structure morphogenesis, carbohydrate metabolism, organic substance metabolism, response to endogenous stimuli, cell wall organization or biosynthesis, carbohydrate derivative metabolism, and general metabolism were significantly down-regulated in response to heat stress (**Table S2**, **S3**, **Figures 4A, B**). Molecular functions associated with unfolded protein binding, misfolded protein binding, heat shock protein binding, RNA binding, HSP90 protein binding, chaperone binding, and ATPase regulator activity were significantly up-regulated. In contrast, phosphotransferase activity, alcohol group as acceptor, secondary active transmembrane transporter activity, anion transmembrane transporter activity, transferase activity, kinase activity, and symporter activity were the most down-regulated molecular functions under heat stress conditions (**Table 2**). Spliceosome, protein processing in the endoplasmic reticulum, and proteasome were the significantly up-regulated pathways in this study. Down-regulated pathways in response to heat stress included biosynthesis of secondary metabolites, metabolic pathways, pentose and glucuronate interconversions, amino sugar and nucleotide sugar metabolism, carbon fixation in photosynthetic organisms, and fatty acid elongation (**Table 3**).

Regarding transcription factors, 128 differentially expressed transcription factors were identified, belonging to 35 families, with 78 being down-expressed and 50 being over-expressed. Among these transcription factor families, bHLH, HSF, ARFs, AP2, TCP, ERF, bZIP, Dof, MYB and MYB-related, C2H2, NAC, and GRAS were the most represented in response to heat stress in this study. Down-expressed transcription factors were detected in the bHLH, ARF, AP2, TCP, MYB, and Dof families, while only over-expressed transcription factors were found in the HSF family.

The AP2 transcription factor family specifically binds to the GCC-box found in the promoters of certain genes. In this family, 30 transcription factors were identified in *A. thaliana*, and four were found to be significantly down-expressed in this study, including RAP2.7 and SMZ, both of which repress the transition to flowering. Heat stress has been shown to cause a reduction in the number of flower buds for many plants (Ali et al., 2020). Perhaps, reducing the expression of these two repressors could be a strategy developed by *A. thaliana* to mitigate the impact of heat on floral development. Further studies are needed to confirm this hypothesis.

Auxin response factor (ARF) is a transcription factor family and specifically binds to the DNA sequence 5’-TGTCTC-3’ located in auxin-responsive promoter elements. Five significantly down-expressed transcription factors were detected belonging to the ARF family, including MP, ETT, ARF16, ARF8, and ARF4. Reducing the expression of some transcription factors involved in activating or repressing auxin-responsive genes alters the cellular response to auxin.

The MYB family contains 168 transcription factors in *A. thaliana* that bind to the DNA in promoter cis-regulatory elements 5’-GGCGCGC-3’ of cell cycle genes. All six significant transcription factors found in this study encoding for the MYB family are down-expressed, including FLP, AT1G49010, AS1, MYB30 (positive regulator of the hypersensitive response induced by pathogens), MYB28 (involved in the upregulation of aliphatic glucosinolate biosynthesis), and MYB16. The bHLH family is the largest family with 225 members. This study highlights seven differentially expressed transcription factors belonging to this family. Similar to ARF, AP2, and MYB transcription factor families, only down-expressed transcription factors were detected in the bHLH family, including SCRM2 (response to deep-freezing), AT1G29950, AT3G07340, AT3G61950 (all involved in regulation of transcription), MYC4 (involved in jasmonic acid gene regulation), BIM1 (positive brassinosteroid-signaling protein), and bHLH071 (possibly involved in stomatal guard cell differentiation).

### 5.3 Heat stress induces the response to several abiotic stresses

Among the differentially expressed genes (DEGs), we observed 61 DEGs linked to response to heat and 73 DEGs linked to the response to temperature stimulus, exhibiting the lowest p-value of less than 3 x 10^-18^ among the enriched biological processes GO terms. These findings emphasize the significance of temperature-related stress in *A. thaliana*’s adaptive mechanisms.

Notably, heat stress is usually associated with oxidative stress and the accumulation of reactive oxygen species in plants (Pucciariello et al., 2012; Fortunato et al., 2023). This could explain the activation of response to oxidative stress and response to hydrogen peroxide biological processes in heat-stressed plants, which help counter the effects of oxidative stress. Additionally, our analysis revealed several other GO terms that highlight the plant’s response to abiotic stimuli, such as heat acclimation, cellular responses to heat and stress, as well as responses to high-light intensity, hypoxia, decreased oxygen levels, and oxygen levels.

Additionally, the Ethylene Responsive Element Binding Factor (ERF) family, with 193 transcription factors in *A. thaliana*, is involved in response to various abiotic stresses (Xie et al., 2019). Nine over-expressed and three down-expressed genes belonging to this family were detected in this study. The over-expressed ERF transcription factors include CRF7, RAP2.6, RAP2.4, DREB19, AT2G40350, DREB2B, RAP2.2, CRF6, and DREB2A, which are involved in various stress responses and plant development; whereas AT4G16750, AT5G07580, and CRF2, the down-expressed ERF transcription factors, are involved in the development of cotyledons, leaves, and embryos.

It is worth mentioning that a previous study reported commonality in biological processes among different stress conditions in *A. thaliana*, including drought, heat, and cold stresses. These shared processes included responses to temperature stimulus and responses to heat (Pathania and Kumar, 2022). This suggests that *A. thaliana* employs overlapping molecular mechanisms to cope with a variety of environmental stresses.

### 5.4 Heat stress increases the repairing protein damage

Heat Shock Proteins (HSPs) and other chaperones play a crucial role in protein-related processes, including proper folding, stabilizing partially unfolded proteins, and preventing unwanted protein aggregation (Park and Seo, 2015). Our meta-analysis revealed the upregulation of several important pathways, molecular functions, and biological processes related to protein processing. Specifically, the upregulated pathways included Protein processing in endoplasmic reticulum and Proteasome. Moreover, we observed various upregulated molecular functions, such as unfolded and misfolded protein binding, heat shock protein binding, misfolded protein binding, Hsp90 protein binding, and chaperone binding. In parallel, the upregulated biological processes included responses to topologically incorrect proteins, protein folding, and cellular responses to unfolded proteins. These findings underscore the significance of these processes in maintaining proper protein structure and function under heat stress conditions.

Additionally, among the 25 HSF transcription factors identified in *A. thaliana*, seven were significantly over-expressed in this study (HSFA2, HSFA1E, HSFA7A, AT-HSFA7B, HSF4, HSFA3, and HSFB2A) (**Table 4**). HSFA2 is involved in the acquisition of heat memory. It has been shown that *hsfa*2 knockout *A. thaliana* exhibits a faster decline in heat shock protein (HSP) expression in response to heat stress compared to the wild type (Lämke et al., 2016). HSFA1E is involved in inducing the expression of HSFA2 (Nishizawa-Yokoi et al., 2011). HSF transcription factors stimulate the expression of heat shock proteins (HSPs), which in turn prevent and repair protein damages (Anckar and Sistonen, 2011). In this study, numerous HSPs were identified as highly over-expressed, such as HSP70b, HSP101, HSP70T-2, HSP17.6II, and HSP70, each with an SES greater than 2. Xu et al. (2018) reported the over-expression of HSP70 in all analyzed fine fescue cultivars under heat stress conditions. The over-expression of these proteins is aimed at repairing protein damage caused by heat stress, which also explains the activation of chaperon-mediated protein folding biological pathway, unfolded protein binding molecular function, heat shock protein binding molecular function, and chaperone binding molecular function.

### 5.5 Heat stress induces alternative splicing

It becomes evident that heat stress exerts a significant impact on gene regulation in plants, not only at the transcriptional level but also through post-transcriptional mechanisms, particularly alternative splicing. Alternative splicing increases the diversity of functional proteins by generating multiple mRNA products from a single pre-mRNA transcript (Xue et al., 2023). The meta-analysis conducted on *A. thaliana* in this study identified 46 upregulated DEGs associated with RNA splicing under heat stress conditions, emphasizing the importance of this process in the plant’s response to high temperatures. Notably, several biological processes related to mRNA splicing, (mRNA splicing via spliceosome, RNA splicing via transesterification reactions with bulged adenosine as nucleophile, and RNA splicing via transesterification reactions) exhibited significant upregulation (P-value < 1.2 x 10^-13^), along with the Spliceosome KEGG pathway (P-value < 1.4 x 10^-14^). These findings underscore the role of alternative splicing as a means to diversify the functional proteins generated from a single pre-mRNA transcript under heat stress conditions, a process that seems less pronounced under normal conditions (Laloum et al., 2018). Alternative splicing events have been observed in various plant species in response to heat stress. For instance, in *Brachypodium distachyon*, a total of 1,973 alternative splicing events were identified among 451 differentially expressed genes following exposure to a temperature of 42°C (Chen and Li, 2017). In *Oryza sativa*, the temperature and drought-responsive gene *DREB2B* undergoes alternative splicing. Under normal conditions, exon 2 inclusion results in a non-functional isoform. However, high-temperature exposure leads to exon 2 skipping, forming a functional isoform consisting of exons 1 and 3 (Matsukura et al., 2010). In *Zea mays*, a modest increase in the occurrence of alternatively spliced forms for both ZmHsf04 and ZmHsf17 when subjected to a heat stress treatment at 42°C (Zhang et al., 2020).

### 5.6 Heat stress alters mineral transport

It has been reported that the translocation and accumulation of minerals are severely disrupted under heat-stress conditions (Ali et al., 2020). This may be related to the down-expression of genes involved in mineral transport molecular functions and biological processes. In this study, the most significantly down-regulated molecular functions in response to heat stress included transmembrane transporter activity, transporter activity, ion transmembrane transporter activity, inorganic molecular entity transmembrane transporter activity, and active transmembrane transporter activity (**Table 2**). Ion transmembrane transport was among the most down-regulated biological process subclusters, which include ion transmembrane transport, ion transport, anion transmembrane, establishment of localization, carbohydrate derivative transport, and carbohydrate transport biological processes (**Figure 4B**, **Table S3**).

### 5.7 Heat stress alters fatty acid biosynthesis

In response to heat stress, there was a significant down-regulation of the fatty acid biosynthetic and lipid metabolic pathways, as well as the KEGG pathway responsible for fatty acid elongation, particularly in the production of polyunsaturated fatty acids (as shown in **Table 3**). This could account for the decrease in polyunsaturated fatty acid levels in cellular membranes, a mechanism that increases membrane stability in response to heat stress in plants (Higashi et al., 2015). Polyunsaturated fatty acids are known to be more susceptible to peroxidation (Boca et al., 2014), which can compromise membrane integrity while also increasing membrane fluidity (Los et al., 2013). Consequently, *A. thaliana* adapts to heat stress by decreasing polyunsaturated fatty acid content in its membranes, thus strengthening its ability to withstand the thermal stress more effectively.

### 5.8 Hub genes

Ten hub genes were identified through co-expression network analysis among the list of DEGs. With their ES values greater than 1, very low FDR (less than 0.1%), and consistent differential expression across all 16 selected datasets. These ten genes are inferred to hold pivotal roles in the response to heat stress. They include *At1g63780* (IMP4), *At5g08420*, *At3g12050*, *Atpd*, *At1g53850* (*Pae1*), RH36, *At2g15790* (*Sqn*), EDA14, *At1g12650* (RRP36), and *At3g56990* (*Eda7*). These genes are involved in a diverse array cellular processes, encompassing ribosomal RNA processing, ribosome assembly, and plant development. Their consistent differential expression and significant enrichment values underline their central importance in orchestrating the cellular response to heat stress.

### 5.9 Genes of unknown function

Several genes, including *At4g23493*, *At3g17110*, *At1g27590*, and *At4g17130*, were found to be significantly differentially expressed in this study. However, their specific functions remain unknown, emphasizing the need for further research to elucidate their roles in the biological processes.

## 6 Conclusion

In this study, we conducted a random-effects meta-analysis to investigate the transcriptomic response of *A. thaliana* to heat stress. Our aim was to overcome the limitations of transcription profiling using microarray technology and reveal a more accurate and precise set of differentially expressed genes (DEGs). As a result, we identified 1972 DEGs, including 826 over-expressed and 1146 down-expressed genes. These genes may serve as a resource for potential candidate genes and molecular biomarkers for engineering heat-stress-tolerant plants. The over-expressed genes are primarily involved in heat response and RNA splicing BP, and unfolded protein binding KEGG pathways, while the down-expressed genes are mainly associated with the organization or biogenesis BP, transmembrane transporter activity MF, and secondary metabolite biosynthesis KEGG pathways. Furthermore, we identified 128 differentially expressed transcription factors (TFs) belonging to 35 TF families; co-expression network analysis revealed 10 hub genes.

By providing a comprehensive understanding of the molecular mechanisms involved in heat stress response, this research would serve as a valuable foundation for developing heat-stress-resistant crops, ultimately contributing to global food security in a warming world.

## Data availability statement

The datasets presented in this study can be found in online repositories. The names of the repository/repositories and accession number(s) can be found in the article/**Supplementary Material**.

## Author contributions

ZC contributed to the acquisition and analysis of data, as well as writing the manuscript. KK et IH contributed to data acquisition. AS, KT and MM participated in the revision and validation of the final version of the manuscript. BB designed the work and participated in the writing, revision, and validation of the final manuscript version. All authors contributed to the article and approved the submitted version.
