# Supplementary material for: Meta-analysis of Arabidopsis thaliana microarray data in relation to heat stress response
Source: Front Plant Sci. 2023 Dec 19;14:1250728. doi: 10.3389/fpls.2023.1250728 (PMC10758499; doi:10.3389/fpls.2023.1250728)
Supplement: Supplementary file 1 [file DataSheet_1.docx]

Table S1. Microarray expression profiling datasets for heat stress in *A. thaliana*

*“KO”* Knockout mutant, *“QKO”* quadruple knockout mutant, *“DKO”* double knockout mutant, *“OE”* overexpression, and *“?”* unmentioned information.

| Accession | Database | Year | Platform | Citation | N. of samples without mutant (Control/case) | N. of samples (Control/case) | age (day) | Plant part | Luminosity | Recovery time (hour) | Treated time (min) | T°C case | T°C control | Mutant |
| --- | --- | --- | --- | --- | --- | --- | --- | --- | --- | --- | --- | --- | --- | --- |
| GSE112161 | GEO | 2019 | ATH1-121501 | [PMID: 29884991 (Brzezinka et al., 2019)](https://pubmed.ncbi.nlm.nih.gov/29884991/) | N=9(3/6) | N=18(6/12) | 4 | Whole seedling | ? | 1.5/4/52 | 45/60 | 37/44 | 24 | bru1 KO |
| GSE103398 | GEO | 2018 | ATH1-121501 | [PMID: 29752744 (Hsiang-Chin et al., 2018)](http://europepmc.org/article/MED/29752744) | N=11(3/8) | N=17(5/12) | 4-7 | Whole seedling | ? | 72 | 60 | 37 | 22 | hsfa2 KO |
| GSE83136 | GEO | 2017 | ATH1-121501 | [PMID: 24769482 (Stief et al., 2014)](https://www.ncbi.nlm.nih.gov/pubmed/24769482) | N=9(3/6) | N=18(6/12) | 4 | Whole seedling | ? | 1.5/4/52 | 60/45 | 37/44 | 22 | ago1 KO |
| GSE63372 | GEO | 2016 | ATH1-121501 | [PMID: 27493213 (Huang et al., 2016)](https://www.ncbi.nlm.nih.gov/pubmed/27493213) | N=4(2/2) | N=12(6/6) | 7 | Whole seedling | ? | 0 | 60 | 37 | 22 | HSFA6b OE |
| GSE63128 | GEO | 2015 | ATH1-121501 | [PMID: 26013835 (Higashi et al., 2015)](https://www.ncbi.nlm.nih.gov/pubmed/26013835) | N=27(15/12) | N=27(15/12) | 18-20 | Leaf | Dark/light | 0/8/24 | 480/1440 | 38 | 22 | None |
| GSE58620 | GEO | 2014 | ATH1-121501 | ? | N=6(3/3) | N=6(3/3) | 16-21 | Whole seedling | Dark | 0 | 30 | 37 | 23 | None |
| GSE58616 | GEO | 2014 | ATH1-121501 | ? | N=6(3/3) | N=6(3/3) | 10-15 | Whole seedling | Light | 0 | 30 | 37 | 23 | None |
| GSE44053 | GEO | 2014 | ATH1-121501 | [PMID: 23977042 (Yángüez et al., 2013)](https://www.ncbi.nlm.nih.gov/pubmed/23977042) | N=4(2/2) | N=4(2/2) | 7 | Whole seedling | ? | 0 | 45 | 38 | 22 | None |
| GSE44655 | GEO | 2013 | ATH1-121501 | [PMID: 23832625 (Liu and Charng, 2013)](https://www.ncbi.nlm.nih.gov/pubmed/23832625) | N=4(2/2) | N=16(8/8) | 7 | Whole seedling | ? | 0 | 60 | 37 | 22 | hsfa2 KO/hsfa1 QKO/HSFA2 OE in hsfa1 QKO |
| GSE26197 | GEO | 2011 | ATH1-121501 | [PMID: 21554326 (Weston et al., 2011)](https://www.ncbi.nlm.nih.gov/pubmed/21554326) | N=16(4/12) | N=16(4/12) | ? | Leaf | ? | 0 | 60 | 30/37/39 | 22 | None |
| GSE26266 | GEO | 2010 | ATH1-121501 | [PMID: 22516818 (Shedge et al., 2010)](https://www.ncbi.nlm.nih.gov/pubmed/22516818) | N=4(2/2) | N=12(4/8) | 7 | Whole seedling | ? | 0 | 60 | 37 | 22 | bZIP28 KO |
| GSE19603 | GEO | 2010 | ATH1-121501 | [PMID: 20139171 (Liu and Charng, 2012)](https://www.ncbi.nlm.nih.gov/pubmed/20139171) | N=5(2/3) | N=12(6/6) | 58 | Shoots | ? | 0 | 48 | 37 | 24 | msh1/recA3 DKO |
| GSE12619 | GEO | 2009 | ATH1-121501 | [PMID: 19302169 (Chi et al., 2009)](https://www.ncbi.nlm.nih.gov/pubmed/19302169) | N=4(2/2) | N=8(4/4) | 7 | Whole seedling | ? | 0 | 60 | 37 | 22 | til1-1 KO |
| GSE16222 | GEO | 2009 | ATH1-121501 | [PMID: 20089772 (Banti et al., 2010)](https://www.ncbi.nlm.nih.gov/pubmed/20089772) | N=4(2/2) | N=4(2/2) | 4 | Whole seedling | Dark | 0 | 90 | 38 | 23 | None |
| GSE6154^^[[1]](#footnote-2)^^ | GEO | 2007 | ATH1-121501 | ? | N=3(1/2) | N=6(2/4) | ? | ? | ? | 0 | 60 | 30/40 | 20 | AtrbohB KO |
| GSE4760 | GEO | 2006 | ATH1-121501 | [PMID: 17085506 (Charng et al., 2007)](https://www.ncbi.nlm.nih.gov/pubmed/17085506) | N=6(2/4) | N=12(4/8) | 15 | Whole seedling | Dark | 0/48 | 60/45 | 37/44 | 24 | hsfA2-1 KO |
| GSE4062 | GEO | 2006 | ATH1-121501 | [PMID: 16500991 (Charng et al., 2006)](https://www.ncbi.nlm.nih.gov/pubmed/16500991) | N=4(2/2) | N=8(4/4) | 15 | Shoots | Dark | 0 | 120 | 37 | 22 | hsa32 KO |
| E-MEXP-1725 | AE | 2008 | ATH1-121501 | [PMID:19529832 (Kumar et al., 2009)](http://europepmc.org/abstract/MED/19529832) | N=4(2/2) | N=8(4/4) | 35 | Leaf | ? | 0 | 120 | 37 | 22 | hsfB1/B2b DKO |
| E-MEXP-98 | AE | 2004 | ATH1-121501 | [PMID:15610345 (Busch et al., 2005)](http://europepmc.org/abstract/MED/15610345) | N=4(2/2) | N=8(4/4) | 38 | Leaf | Dark | 0 | 60 | 37 | 20 | hsfA1a/hsfA1b DKO |

- Table S2. Biological process gene ontology (GO) enrichment analysis performed using g: Profiler database for the over-expressed genes with a significance p-value < 0.05%.

| GO BP Term name | Term id | Adjusted p value | Term size | Intersection size |
| --- | --- | --- | --- | --- |
| response to heat | GO:0009408 | 1.51E-34 | 244 | 61 |
| protein folding | GO:0006457 | 1.19E-19 | 193 | 41 |
| response to temperature stimulus | GO:0009266 | 3.24E-18 | 656 | 73 |
| mRNA splicing, via spliceosome | GO:0000398 | 1.42E-14 | 236 | 39 |
| RNA splicing, via transesterification reactions with bulged adenosine as nucleophile | GO:0000377 | 8.23E-14 | 261 | 40 |
| RNA splicing, via transesterification reactions | GO:0000375 | 8.23E-14 | 261 | 40 |
| RNA splicing | GO:0008380 | 1.26E-13 | 347 | 46 |
| cellular response to topologically incorrect protein | GO:0035967 | 4.17E-13 | 82 | 23 |
| response to topologically incorrect protein | GO:0035966 | 3.00E-12 | 89 | 23 |
| response to abiotic stimulus | GO:0009628 | 3.16E-11 | 2372 | 141 |
| heat acclimation | GO:0010286 | 9.60E-11 | 58 | 18 |
| cellular response to heat | GO:0034605 | 6.42E-10 | 82 | 20 |
| RNA processing | GO:0006396 | 7.49E-10 | 979 | 75 |
| mRNA processing | GO:0006397 | 1.34E-09 | 479 | 48 |
| cellular response to unfolded protein | GO:0034620 | 1.34E-09 | 58 | 17 |
| response to unfolded protein | GO:0006986 | 1.34E-09 | 58 | 17 |
| cellular response to stress | GO:0033554 | 1.38E-09 | 1329 | 91 |
| chaperone-mediated protein folding | GO:0061077 | 2.66E-09 | 69 | 18 |
| response to high light intensity | GO:0009644 | 5.16E-09 | 81 | 19 |
| mRNA metabolic process | GO:0016071 | 1.57E-08 | 623 | 54 |
| response to hydrogen peroxide | GO:0042542 | 1.17E-07 | 85 | 18 |
| 'de novo' protein folding | GO:0006458 | 2.68E-07 | 59 | 15 |
| cellular response to hypoxia | GO:0071456 | 5.01E-07 | 239 | 29 |
| protein refolding | GO:0042026 | 5.24E-07 | 36 | 12 |
| cellular response to decreased oxygen levels | GO:0036294 | 6.10E-07 | 241 | 29 |
| cellular response to oxygen levels | GO:0071453 | 6.10E-07 | 241 | 29 |
| response to hypoxia | GO:0001666 | 1.24E-06 | 264 | 30 |
| response to decreased oxygen levels | GO:0036293 | 1.77E-06 | 268 | 30 |
| response to oxygen levels | GO:0070482 | 1.94E-06 | 269 | 30 |
| chaperone cofactor-dependent protein refolding | GO:0051085 | 4.08E-06 | 51 | 13 |
| 'de novo' posttranslational protein folding | GO:0051084 | 4.08E-06 | 51 | 13 |
| response to endoplasmic reticulum stress | GO:0034976 | 5.07E-06 | 106 | 18 |
| protein transport | GO:0015031 | 1.11E-05 | 874 | 60 |
| intracellular protein transport | GO:0006886 | 1.41E-05 | 629 | 48 |
| establishment of protein localization | GO:0045184 | 1.83E-05 | 886 | 60 |
| ERAD pathway | GO:0036503 | 2.03E-05 | 68 | 14 |
| peptide transport | GO:0015833 | 3.48E-05 | 924 | 61 |
| intracellular transport | GO:0046907 | 4.29E-05 | 799 | 55 |
| response to light intensity | GO:0009642 | 6.03E-05 | 166 | 21 |
| ubiquitin-dependent ERAD pathway | GO:0030433 | 6.87E-05 | 53 | 12 |
| amide transport | GO:0042886 | 7.45E-05 | 944 | 61 |
| protein-containing complex subunit organization | GO:0043933 | 0.00015 | 830 | 55 |
| ubiquitin-dependent protein catabolic process | GO:0006511 | 0.000199 | 501 | 39 |
| nitrogen compound transport | GO:0071705 | 0.000209 | 1226 | 72 |
| positive regulation of biological process | GO:0048518 | 0.000343 | 1531 | 84 |
| establishment of localization in cell | GO:0051649 | 0.000418 | 857 | 55 |
| modification-dependent protein catabolic process | GO:0019941 | 0.000423 | 516 | 39 |
| response to reactive oxygen species | GO:0000302 | 0.000465 | 187 | 21 |
| response to oxidative stress | GO:0006979 | 0.00047 | 498 | 38 |
| positive regulation of nucleobase-containing compound metabolic process | GO:0045935 | 0.000488 | 686 | 47 |
| modification-dependent macromolecule catabolic process | GO:0043632 | 0.000827 | 530 | 39 |
| response to inorganic substance | GO:0010035 | 0.000887 | 1015 | 61 |
| positive regulation of macromolecule metabolic process | GO:0010604 | 0.000948 | 948 | 58 |
| response to stress | GO:0006950 | 0.001028 | 3969 | 174 |
| positive regulation of RNA metabolic process | GO:0051254 | 0.001108 | 663 | 45 |
| protein localization | GO:0008104 | 0.001251 | 1049 | 62 |
| cellular protein localization | GO:0034613 | 0.00136 | 800 | 51 |
| protein-containing complex assembly | GO:0065003 | 0.001386 | 734 | 48 |
| positive regulation of gene expression | GO:0010628 | 0.001429 | 691 | 46 |
| nucleobase-containing compound metabolic process | GO:0006139 | 0.002241 | 4959 | 207 |
| cellular macromolecule localization | GO:0070727 | 0.002276 | 837 | 52 |
| regulation of mRNA splicing, via spliceosome | GO:0048024 | 0.002919 | 50 | 10 |
| positive regulation of nitrogen compound metabolic process | GO:0051173 | 0.003016 | 914 | 55 |
| RNA metabolic process | GO:0016070 | 0.003022 | 4037 | 174 |
| response to chemical | GO:0042221 | 0.003724 | 3407 | 151 |
| endoplasmic reticulum unfolded protein response | GO:0030968 | 0.004079 | 41 | 9 |
| proteolysis involved in cellular protein catabolic process | GO:0051603 | 0.004797 | 613 | 41 |
| proteasomal protein catabolic process | GO:0010498 | 0.00548 | 328 | 27 |
| positive regulation of metabolic process | GO:0009893 | 0.006211 | 1031 | 59 |
| positive regulation of transcription from RNA polymerase II promoter in response to heat stress | GO:0061408 | 0.0065 | 24 | 7 |
| positive regulation of transcription from RNA polymerase II promoter in response to stress | GO:0036003 | 0.0065 | 24 | 7 |
| cellular protein catabolic process | GO:0044257 | 0.006824 | 622 | 41 |
| protein catabolic process | GO:0030163 | 0.007198 | 712 | 45 |
| gene expression | GO:0010467 | 0.007448 | 4554 | 190 |
| ATP metabolic process | GO:0046034 | 0.007661 | 171 | 18 |
| positive regulation of nucleic acid-templated transcription | GO:1903508 | 0.00892 | 629 | 41 |
| positive regulation of RNA biosynthetic process | GO:1902680 | 0.00892 | 629 | 41 |
| positive regulation of macromolecule biosynthetic process | GO:0010557 | 0.010975 | 679 | 43 |
| protein deneddylation | GO:0000338 | 0.012293 | 11 | 5 |
| nucleic acid metabolic process | GO:0090304 | 0.012577 | 4563 | 189 |
| positive regulation of cellular process | GO:0048522 | 0.014262 | 1280 | 68 |
| regulation of transcription from RNA polymerase II promoter in response to stress | GO:0043618 | 0.01537 | 27 | 7 |
| cellular localization | GO:0051641 | 0.015816 | 1088 | 60 |
| regulation of mRNA processing | GO:0050684 | 0.016174 | 60 | 10 |
| positive regulation of cellular metabolic process | GO:0031325 | 0.017289 | 971 | 55 |
| cellular respiration | GO:0045333 | 0.017465 | 148 | 16 |
| response to organonitrogen compound | GO:0010243 | 0.017527 | 291 | 24 |
| regulation of RNA splicing | GO:0043484 | 0.01881 | 61 | 10 |
| alternative mRNA splicing, via spliceosome | GO:0000380 | 0.019139 | 49 | 9 |
| regulation of mRNA metabolic process | GO:1903311 | 0.0195 | 88 | 12 |
| regulation of DNA-templated transcription in response to stress | GO:0043620 | 0.019943 | 28 | 7 |
| positive regulation of transcription, DNA-templated | GO:0045893 | 0.023662 | 611 | 39 |
| mRNA cis splicing, via spliceosome | GO:0045292 | 0.024103 | 39 | 8 |
| cellular component assembly | GO:0022607 | 0.025558 | 1033 | 57 |
| cellular response to chemical stimulus | GO:0070887 | 0.027706 | 1793 | 87 |
| regulation of alternative mRNA splicing, via spliceosome | GO:0000381 | 0.02931 | 40 | 8 |
| positive regulation of cellular biosynthetic process | GO:0031328 | 0.029842 | 708 | 43 |
| response to light stimulus | GO:0009416 | 0.030606 | 778 | 46 |
| protein modification by small protein removal | GO:0070646 | 0.030751 | 92 | 12 |
| spindle disassembly | GO:0051230 | 0.032237 | 3 | 3 |
| mitotic spindle disassembly | GO:0051228 | 0.032237 | 3 | 3 |
| regulation of ATPase activity | GO:0043462 | 0.032475 | 30 | 7 |
| heme transport | GO:0015886 | 0.032507 | 13 | 5 |
| cellular macromolecule catabolic process | GO:0044265 | 0.039293 | 833 | 48 |
| organic substance transport | GO:0071702 | 0.039666 | 1500 | 75 |
| proteasome-mediated ubiquitin-dependent protein catabolic process | GO:0043161 | 0.039678 | 306 | 24 |
| proteolysis | GO:0006508 | 0.041303 | 1174 | 62 |
| heterocycle metabolic process | GO:0046483 | 0.043074 | 5300 | 211 |
| cellular nitrogen compound metabolic process | GO:0034641 | 0.045398 | 6016 | 235 |

- Table S3. Biological process gene ontology (GO) enrichment analysis performed using g: Profiler database for the down-expressed genes with a significance p-value < 0.05%.

| GO BP Term name | Term id | Adjusted p value | Term size | Intersection size |
| --- | --- | --- | --- | --- |
| transmembrane transport | GO:0055085 | 2.24E-12 | 1343 | 124 |
| carbohydrate metabolic process | GO:0005975 | 5.00E-10 | 1221 | 110 |
| cell wall organization or biogenesis | GO:0071554 | 2.02E-08 | 775 | 77 |
| ion transport | GO:0006811 | 2.81E-08 | 1075 | 96 |
| protein phosphorylation | GO:0006468 | 8.00E-08 | 1292 | 108 |
| ion transmembrane transport | GO:0034220 | 2.34E-06 | 718 | 68 |
| cell wall organization | GO:0071555 | 4.86E-06 | 609 | 60 |
| establishment of localization | GO:0051234 | 6.25E-06 | 2878 | 189 |
| polysaccharide metabolic process | GO:0005976 | 1.09E-05 | 518 | 53 |
| secondary metabolite biosynthetic process | GO:0044550 | 1.45E-05 | 176 | 27 |
| small molecule metabolic process | GO:0044281 | 2.41E-05 | 1897 | 134 |
| phosphorylation | GO:0016310 | 2.57E-05 | 1754 | 126 |
| localization | GO:0051179 | 2.61E-05 | 3107 | 198 |
| pectin metabolic process | GO:0045488 | 3.43E-05 | 195 | 28 |
| transport | GO:0006810 | 3.78E-05 | 2832 | 183 |
| galacturonan metabolic process | GO:0010393 | 3.84E-05 | 196 | 28 |
| external encapsulating structure organization | GO:0045229 | 5.21E-05 | 650 | 60 |
| fatty acid biosynthetic process | GO:0006633 | 7.12E-05 | 166 | 25 |
| carbohydrate derivative metabolic process | GO:1901135 | 9.83E-05 | 871 | 73 |
| cation transport | GO:0006812 | 0.000136 | 668 | 60 |
| cellular process | GO:0009987 | 0.000156 | 16066 | 780 |
| small molecule biosynthetic process | GO:0044283 | 0.000166 | 768 | 66 |
| anion transport | GO:0006820 | 0.000179 | 429 | 44 |
| monocarboxylic acid biosynthetic process | GO:0072330 | 0.00021 | 277 | 33 |
| phosphorus metabolic process | GO:0006793 | 0.000219 | 2537 | 164 |
| carbohydrate biosynthetic process | GO:0016051 | 0.000326 | 380 | 40 |
| plant-type cell wall organization or biogenesis | GO:0071669 | 0.000364 | 311 | 35 |
| organic acid metabolic process | GO:0006082 | 0.000387 | 1299 | 96 |
| anatomical structure morphogenesis | GO:0009653 | 0.000422 | 972 | 77 |
| sulfur compound biosynthetic process | GO:0044272 | 0.000481 | 183 | 25 |
| oxoacid metabolic process | GO:0043436 | 0.000521 | 1290 | 95 |
| plant organ development | GO:0099402 | 0.000587 | 1066 | 82 |
| organic substance metabolic process | GO:0071704 | 0.00077 | 11945 | 601 |
| protein autophosphorylation | GO:0046777 | 0.000888 | 254 | 30 |
| carboxylic acid biosynthetic process | GO:0046394 | 0.000978 | 580 | 52 |
| organic acid biosynthetic process | GO:0016053 | 0.000978 | 580 | 52 |
| carboxylic acid biosynthetic process | GO:0046394 | 0.000978 | 580 | 52 |
| lipid metabolic process | GO:0006629 | 0.001106 | 1153 | 86 |
| anion transmembrane transport | GO:0098656 | 0.001319 | 232 | 28 |
| shoot system morphogenesis | GO:0010016 | 0.001589 | 195 | 25 |
| inorganic ion transmembrane transport | GO:0098660 | 0.001689 | 512 | 47 |
| phenylpropanoid biosynthetic process | GO:0009699 | 0.001784 | 101 | 17 |
| fatty acid metabolic process | GO:0006631 | 0.002351 | 280 | 31 |
| carbohydrate derivative transport | GO:1901264 | 0.003186 | 128 | 19 |
| very long-chain fatty acid metabolic process | GO:0000038 | 0.003562 | 23 | 8 |
| carboxylic acid metabolic process | GO:0019752 | 0.004464 | 1123 | 82 |
| phosphate-containing compound metabolic process | GO:0006796 | 0.004896 | 2494 | 155 |
| response to endogenous stimulus | GO:0009719 | 0.007454 | 1877 | 122 |
| plant organ morphogenesis | GO:1905392 | 0.008193 | 417 | 39 |
| plant-type cell wall organization | GO:0009664 | 0.008236 | 174 | 22 |
| cation transmembrane transport | GO:0098655 | 0.008583 | 496 | 44 |
| polysaccharide biosynthetic process | GO:0000271 | 0.008786 | 228 | 26 |
| very long-chain fatty acid biosynthetic process | GO:0042761 | 0.009497 | 19 | 7 |
| metabolic process | GO:0008152 | 0.009923 | 13480 | 659 |
| root development | GO:0048364 | 0.01042 | 548 | 47 |
| carbohydrate derivative biosynthetic process | GO:1901137 | 0.010483 | 532 | 46 |
| root system development | GO:0022622 | 0.010924 | 549 | 47 |
| response to hormone | GO:0009725 | 0.011946 | 1839 | 119 |
| post-embryonic root development | GO:0048528 | 0.014819 | 142 | 19 |
| primary metabolic process | GO:0044238 | 0.020062 | 11236 | 559 |
| inorganic anion transport | GO:0015698 | 0.02432 | 147 | 19 |
| monocarboxylic acid metabolic process | GO:0032787 | 0.024856 | 567 | 47 |
| inorganic cation transmembrane transport | GO:0098662 | 0.025327 | 454 | 40 |
| plant organ formation | GO:1905393 | 0.029821 | 124 | 17 |
| monovalent inorganic cation transport | GO:0015672 | 0.030472 | 318 | 31 |
| carbohydrate transport | GO:0008643 | 0.04265 | 153 | 19 |
| metal ion transport | GO:0030001 | 0.043235 | 417 | 37 |
| cellular carbohydrate metabolic process | GO:0044262 | 0.043824 | 514 | 43 |
| nucleotide-sugar biosynthetic process | GO:0009226 | 0.045637 | 40 | 9 |
| lateral root development | GO:0048527 | 0.049804 | 129 | 17 |


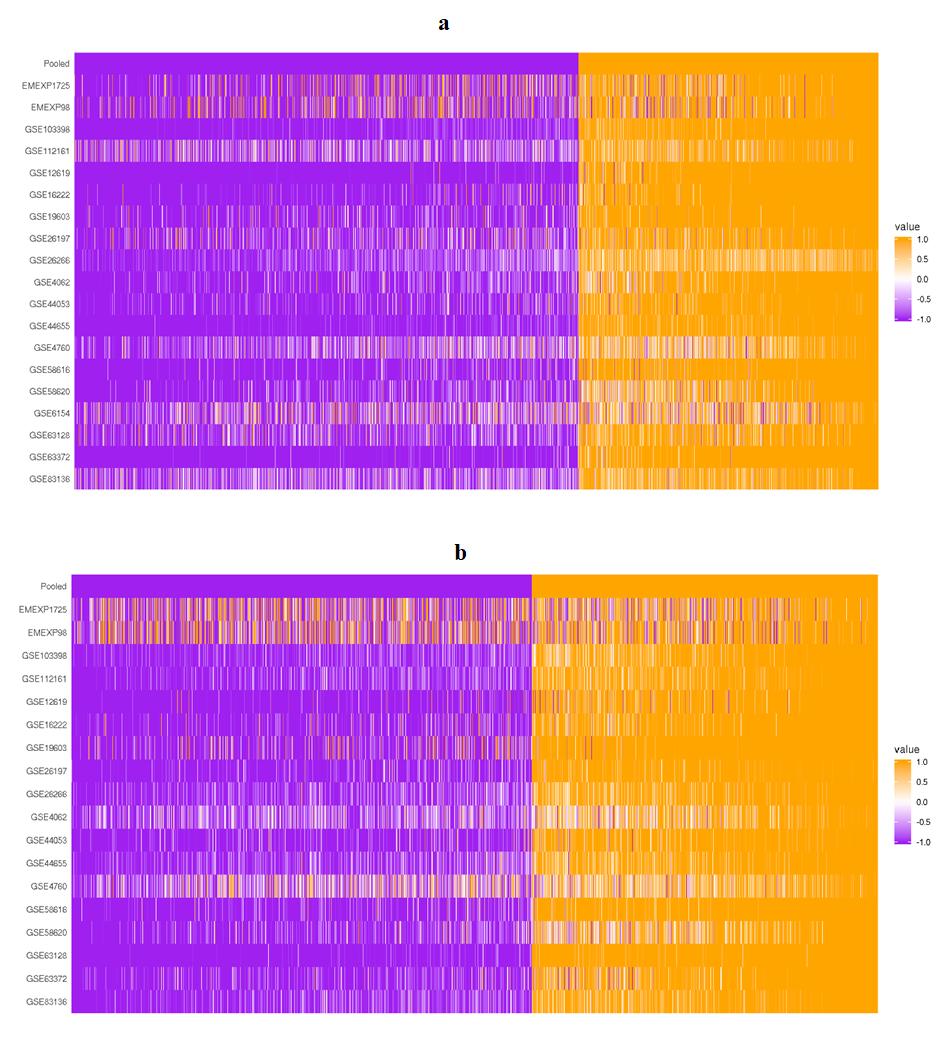


Figure S1. Heatmap for the selected DEGs (including heterogeneous DEGs) for the first meta-analysis (with keeping mutant samples) (a) and in the second meta-analysis (with removing all mutant samples) (b).


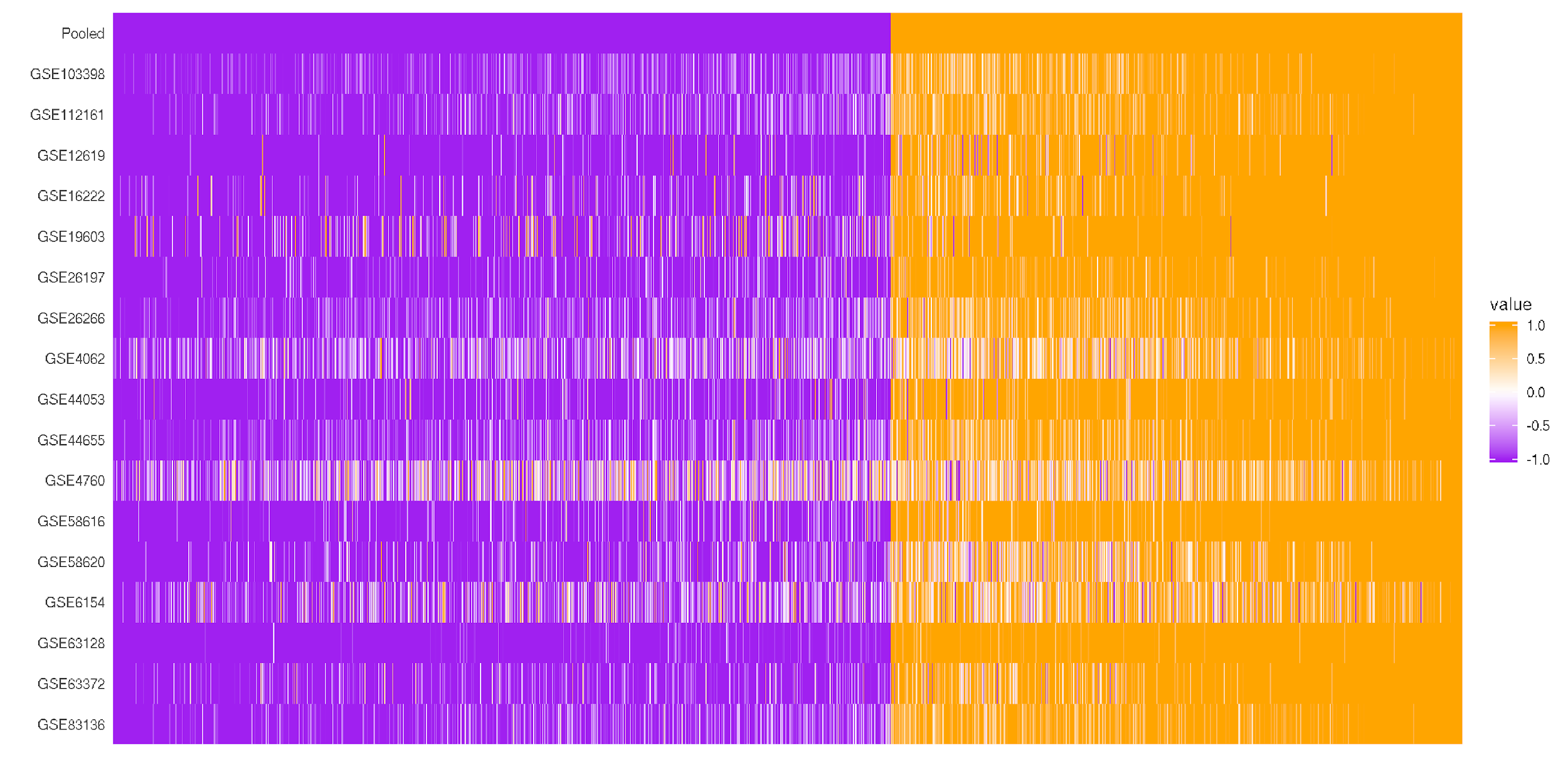
Figure S2: Heatmap generated for the obtained 1972 DEGs in the meta-analysis of the 16 datasets with an effect size threshold of 1, an FDR of 0.001, and a heterogeneity cutoff of 0.05.

**References**

Banti, V., Mafessoni, F., Loreti, E., Alpi, A., and Perata, P. (2010). The heat-inducible transcription factor HsfA2 enhances anoxia tolerance in *Arabidopsis*. Plant Physiol. 152, 1471–1483. doi: 10.1104/pp.109.149815

Brzezinka, K., Altmann, S., and Bäurle, I. (2019). BRUSHY1/TONSOKU/MGOUN3 is required for heat stress memory. Plant Cell Environ. 42, 771–781. doi: 10.1111/pce.13365

Busch, W., Wunderlich, M., and Schöfﬂ, F. (2005). Identiﬁcation of novel heat shock factor-dependent genes and biochemical pathways in *Arabidopsis thaliana*. Plant J. 41, 1–14. doi: 10.1111/j.1365-313X.2004.02272.x

Charng, Y., Liu, H., Liu, N., Chi, W., Wang, C., Chang, S., et al. (2007). A heat- inducible transcription factor, *hsfA2*, is required for extension of acquired thermotolerance in *Arabidopsis*. Plant Physiol. 143, 251–262. doi: 10.1104/ pp.106.091322

Charng, Y., Liu, H., Liu, N., Hsu, F., and Ko, S. (2006). *Arabidopsis* Hsa32, a Novel Heat Shock Protein, Is Essential for Acquired Thermotolerance during Long Recovery after Acclimation. Plant Physiol. 140, 1297–1305. doi: 10.1104/pp.105.074898

Chi, W.-T., Fung, R. W. M., Liu, H.-C., Hsu, C.-C., and Charng, Y.-Y. (2009). Temperature-induced lipocalin is required for basal and acquired thermotolerance in *Arabidopsis*. Plant Cell Environ. 32, 917–927. doi: 10.1111/j.1365-3040.2009.01972.x

Huang, Y.-C., Niu, C.-Y., Yang, C.-R., and Jinn, T.-L. (2016). The heat stress factor HSFA6b connects ABA signaling and ABA-mediated heat responses. Plant Physiol. 172, 1182–1199. doi: 10.1104/pp.16.00860

Liu, H., and Charng, Y. (2012). Acquired thermotolerance independent of heat shock factor A1 (HsfA1), the master regulator of the heat stress response. Plant Signaling Behav. 7, 547–550. doi: 10.4161/psb.19803

Liu, H., and Charng, Y. (2013). Common and distinct functions of *Arabidopsis* class A1 and A2 heat shock factors in diverse abiotic stress responses and development. Plant Physiol. 163, 276–290. doi: 10.1104/pp.113.221168

Shedge, V., Davila, J., Arrieta-Montiel, M. P., Mohammed, S., and Mackenzie, S. A. (2010). Extensive rearrangement of the *Arabidopsis* mitochondrial genome elicits cellular conditions for thermotolerance. Plant Physiol. 152 (4), 1960–1970. doi: 10.1104/pp.109.152827

Stief, A., Altmann, S., Hoffmann, K., Pant, B. D., Scheible, W.-R., and Bäurle, I. (2014). *Arabidopsis* miR156 Regulates Tolerance to Recurring Environmental Stress through SPL Transcription Factors. Plant Cell. 26, 1792–1807. doi: 10.1105/tpc.114.123851

Weston, D. J., Karve, A. A., Gunter, L. E., Jawdy, S. S., Yang, X., Allen, S. M., et al. (2011). Comparative physiology and transcriptional networks underlying the heat shock response in Populus trichocarpa, *Arabidopsis thaliana* and Glycine max. Plant Cell Environ. 34, 1488–1506. doi: 10.1111/j.1365-3040.2011.02347.x

Yá ngüez, E., Castro-Sanz, A. B., Ferná ndez-Bautista, N., Oliveros, J. C., and Castellano, M. M. (2013). Analysis of genome-wide changes in the translatome of *Arabidopsis* seedlings subjected to heat stress. PloS One 8, e71425. Doi: 10.1371/journal.pone.0071425

1. Dataset GSE6154 was omitted to perform a meta-analysis without mutant samples due to the number of control samples after the removal of mutant samples. [↑](#footnote-ref-2)
